# GitHub Statistics as a Measure of the Impact of Open-Source Bioinformatics Software

**DOI:** 10.3389/fbioe.2018.00198

**Published:** 2018-12-18

**Authors:** Mikhail G. Dozmorov

**Affiliations:** Department of Biostatistics, Virginia Commonwealth University, Richmond, VA, United States

**Keywords:** bioinformatics, software, impact factor, altmetrics, github

## Abstract

Modern research is increasingly data-driven and reliant on bioinformatics software. Publication is a common way of introducing new software, but not all bioinformatics tools get published. Giving there are competing tools, it is important not merely to find the appropriate software, but have a metric for judging its usefulness. Journal's impact factor has been shown to be a poor predictor of software popularity; consequently, focusing on publications in high-impact journals limits user's choices in finding useful bioinformatics tools. Free and open source software repositories on popular code sharing platforms such as GitHub provide another venue to follow the latest bioinformatics trends. The open source component of GitHub allows users to bookmark and copy repositories that are most useful to them. This Perspective aims to demonstrate the utility of GitHub “stars,” “watchers,” and “forks” (GitHub statistics) as a measure of software impact. We compiled lists of impactful bioinformatics software and analyzed commonly used impact metrics and GitHub statistics of 50 genomics-oriented bioinformatics tools. We present examples of community-selected best bioinformatics resources and show that GitHub statistics are distinct from the journal's impact factor (JIF), citation counts, and alternative metrics (Altmetrics, CiteScore) in capturing the level of community attention. We suggest the use of GitHub statistics as an unbiased measure of the usability of bioinformatics software complementing the traditional impact metrics.

## Introduction

It is currently undeniable that bioinformatics tools and databases represent a highly impactful part of modern research (Wren, 2016). Many journals focus exclusively on publishing software tools and databases. Some of the most famous examples include application notes published in *Bioinformatics*, database, and web-server issues published by *Nucleic Acids Research*, software articles published in *Frontiers Bioinformatics and Computational Biology, PLOS Computational Biology, BMC Bioinformatics*. However, given the continued growth of bioinformatics publications (Wren, 2016) (Supplementary Figure 1), it is getting increasingly difficult to find software that will be useful in real-life applications. Recently, a term “software crisis” was coined to illustrate the problem of finding useful software (Mangul et al., 2018).

Finding useful bioinformatics software is further hindered by publication lag. It often takes more than a year from the time of presubmission inquiry, potential resubmission and the peer-review period to the accepted publication. Such delays inevitably diminish the potential impact of published software. Non-peer-reviewed preprint publishing (arXiv, biorXiv, PeerJ, AsapBio) aims to eliminate publication lag. However, the number of preprints grows nearly 10 times faster than the number of peer-reviewed publications^1^, ^2^ further complicating finding useful software.

Reviews of bioinformatics resources can help orient a scientist in the wealth of published tools and databases. Such reviews are typically written about bioinformatics software published in high-impact journals while leaving preprints and unpublished software largely out of scope. Furthermore, reviews may be limited by the experience of the authors, as well as by a bias to review software published in high-impact journals. Thus, while helpful in orienting a novice in the topic, reviews may overlook useful bioinformatics resources.

Although the peer-review process helps to publish high-quality bioinformatics software, it is unknown at the time of publication which tools and databases will be embraced by the scientific community and which will be forgotten (Wren and Bateman, 2008). In fact, a study based on text mining found that over 70% of published bioinformatics software resources are never reused (Duck et al., 2016). A recent analysis of the usability of bioinformatics software confirmed these observations by highlighting issues with software accessibility and installation (Mangul et al., 2018). Notably, a journal's impact factor, calculated as the average number of citations received in a calendar year by the total number of articles and reviews published in that journal in the preceding 2 years (JIF) is not a good predictor of software popularity (Seglen, 1997; Wren, 2016), making it hard to predict whether a bioinformatics tool or a database published in a high-impact journal will be useful in real-life applications.

## Limitations of Alternative Metrics to Measure the Impact of Bioinformatics Software

Alternative metrics have been proposed to alleviate the shortcomings of JIF or the lack of it in preprint publishing. CiteScore, a metric developed by Scopus includes more document types and citation sources, and uses the 3-year time window to calculate the ratio of citations over the total number of citable items, has been proposed as a consistent alternative to JIF (Silva and Memon, 2017). Article-level metrics, or Altmetrics, is currently the most widely used alternative to measure the impact of scholarly material, including preprints (Priem et al., 2010; Shema et al., 2014). In addition to academic citations, this metric aggregates mentions in social media networks, such as Twitter, online discussions, and recommendations. Although in principle Altmetrics can be applied to any research output that has a digital object identifier (DOI), including datasets, code, and software (Piwowar, 2013), its use for measuring the impact of bioinformatics software is less common. Furthermore, Altmetrics may still be biased by high impact factor (hence, greater exposure, and discussion) (Adie, 2013), and overlook the practical usability of software. The usefulness of these alternative metrics on measuring the impact of bioinformatics software remains unknown.

## Community-guided Selection of Bioinformatics Resources

An increasing number of bioinformaticians choose to develop their tools on popular code sharing web services, such as GitHub (Wilson et al., 2017). Besides code-sharing services, GitHub combines a version control system (Bryan, 2017) with features found in popular social network sites such as Facebook and Twitter (Lima et al., 2014). Users may try the tools and bookmark the most practically useful ones by “starring,” “watching,” and/or “forking” them. “Starring” a repository is similar to bookmarking it as a favorite, while “watching” is a more advanced feature allowing a user to receive all, or selected, updates about a repository. “Forking” further advances user's involvement by creating a copy of a forked repository under the user's account, allowing him/her to offer code enhancements by creating pull requests. GitHub creates a natural ecosystem for software development where the amount of community attention to a repository is directly proportional to its popularity (Hu et al., 2016). We expect the number of stars, watchers, and forks (“GitHub statistics”) to reflect some evidence of the practical utility of the software and suggest they should be used to inform selection of the most useful resources.

## Lists of Community-selected Software as Reviews of Practical Utility

Although using GitHub statistics as a guide for selecting the most popular software, including bioinformatics tools, has been suggested^3^ (Hu et al., 2016; Russell et al., 2018), it does not alleviate the problem of finding the right field-specific resources among a large number of bioinformatics repositories^4^. The abundance of GitHub repositories gave rise to field-specific collections of the most useful resources (tools, databases, papers, books, and videos), frequently referred to as “awesome” lists (Table 1, Supplementary Table 1). They are assembled by inspired individuals who empirically try them and bookmarks the most valuable repositories (Marlow et al., 2013). These collections of links and notes are themselves published on GitHub and starred by the community. The collections may themselves be assembled into field-specific “awesome” lists of lists (Supplementary Table 2). Being analogous to bookmarks freely accessible on the web, they do not require any programming skills to be used. These collections may be compared with field-specific reviews peer-reviewed by the community and may be used to quickly prioritize practically useful resources.

**Table 1:** Popular collections of bioinformatics resources, accessed on November 30, 2018.

**Name**	**Description**	**URL**	**Stars**	**Watchers**	**Forks**
**GENERAL BIOINFORMATICS COLLECTIONS**					
Deeplearning-biology	A list of deep learning implementations in biology	https://github.com/hussius/deeplearning-biology	775	148	198
Deep-review	A collaboratively written review paper on deep learning genomics and precision medicine	https://github.com/greenelab/deep-review	742	120	188
Awesome-bioinformatics	A curated list of awesome Bioinformatics libraries and software	https://github.com/danielecook/Awesome-Bioinformatics	583	80	158
Awesome	Awesome resources on Bioinformatics data science machine learning programming language Python Golang R Perl and miscellaneous stuff	https://github.com/shenwei356/awesome	304	21	115
Genomicspapers	The Leek group guide to genomics papers	https://github.com/jtleek/genomicspapers	299	54	134
Biotools	A list of useful bioinformatics resources	https://github.com/jdidion/biotools	205	24	60
Getting-started-with-genomics-tools-and-resources	Unix R and python tools for genomics	https://github.com/crazyhottommy/getting-started-with-genomics-tools-and-resources	157	27	69
**FIELD-SPECIFIC BIOINFORMATICS COLLECTIONS**					
Awesome-single-cell	List of software packages for single-cell data analysis including RNA-seq ATAC-seq etc.	https://github.com/seandavi/awesome-single-cell	712	154	303
RNA-seq-analysis	RNAseq analysis notes from Ming Tang	https://github.com/crazyhottommy/RNA-seq-analysis	260	44	104
ChIP-seq-analysis	ChIP-seq analysis notes from Ming Tang	https://github.com/crazyhottommy/ChIP-seq-analysis	252	41	136
Awesome-cancer-variant-databases	A community-maintained repository of cancer clinical knowledge bases and databases focused on cancer variants	https://github.com/seandavi/awesome-cancer-variant-databases	109	23	25
Awesome-10x-genomics	List of tools and resources related to the 10x Genomics GEMCode/Chromium system	https://github.com/johandahlberg/awesome-10x-genomics	63	8	12
DNA-seq-analysis	DNA sequencing analysis notes from Ming Tang	https://github.com/crazyhottommy/DNA-seq-analysis	53	7	34
Awesome-microbes	List of computational resources for analyzing microbial sequencing data	https://github.com/stevetsa/awesome-microbes	33	5	16
DNA-methylation-analysis	DNA methylation analysis notes from Ming Tang	https://github.com/crazyhottommy/DNA-methylation-analysis	25	4	22

## Community Attention as a Distinct and Universal Measure of Software Impact

**Figure 1:**
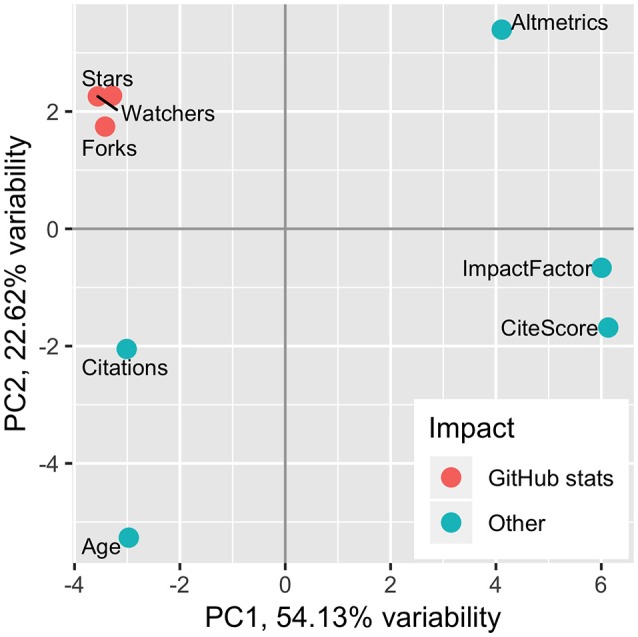
Principal component analysis of bioinformatics impact measures, colored by metric type.

To better understand the relationship between community attention-based and traditional impact metrics, we compared GitHub statistics, JIF, CiteScore, Altmetrics, citation count, and software age of 50 popular genomics-oriented bioinformatics tools published in peer-review journals, developed on GitHub, and starred 50 times or more (Supplementary Table 3, Methods^5^). Principal component analysis (PCA, Figure 1) and correlation analysis (Supplementary Figure 2) showed the expected correlation between similarly calculated JIF and CiteScore (Pearson Correlation Coefficient, PCC = 0.73). The software age and citation counts were also correlated (PCC = 0.60) as would be expected for older software having more chance of being cited. However, neither the software age nor citation counts were correlated with JIF (PCC = −0.23/−0.02, respectively), suggesting that citations of bioinformatics software have minimal effect on JIF. Furthermore, the correlation between JIF and Altmetrics was relatively modest (PCC = 0.49), suggesting that Altmetrics captures a different level of impact. The poor correlation among traditional impact metrics complicates their use for measuring the software impact.

Being a measure of attention of open-source software development community, GitHub statistics are expected to capture the practical usability of software that may be missed by traditional impact metrics. Indeed, GitHub statistics (counts of “stars,” “watches,” and “forks”) were highly correlated with each other (average PCC = 0.92) but were distinct from other metrics. Neither JIF nor Altmetrics correlated with GitHub statistics (average PCC = −0.09/0.14, respectively), highlighting differences between community attention-based and traditional impact metrics. Interestingly, GitHub statistics and citation counts showed modest correlation (average PCC = 0.66), suggesting that practically useful software cited more frequently. However, the software age correlated with GitHub statistics to a much lesser extent (average PCC = 0.32), suggesting that the age of the software does not necessarily indicate its usefulness. We suggest that GitHub statistics should be used as an objective addition to JIF and other traditional impact metrics in measuring the practical utility of bioinformatics software.

## Author Contributions

MD envisioned the project, collected and analyzed the data, and wrote the manuscript.

### Conflict of Interest Statement

The author declares that the research was conducted in the absence of any commercial or financial relationships that could be construed as a potential conflict of interest.

## Footnotes

^1^https://www.crossref.org/blog/preprints-growth-rate-ten-times-higher-than-journal-articles/

^2^http://www.prepubmed.org/monthly_stats/?Subject=Bioinformatics

^3^https://gitstar-ranking.com/

^4^https://www.researchgate.net/post/Is_there_too_many_bioinformatics_tools2

^5^The data and the methods are available at https://github.com/mdozmorov/bioinformatics-impact
