# Supplementary material for: GitHub Statistics as a Measure of the Impact of Open-Source Bioinformatics Software
Source: Front Bioeng Biotechnol. 2018 Dec 18;6:198. doi: 10.3389/fbioe.2018.00198 (PMC6306043; doi:10.3389/fbioe.2018.00198)
Supplement: Supplementary file 1 [file Data_Sheet_1.PDF]

Supplementary Material

Impact of bioinformatics software: community votes as a usability metric

Mikhail G. Dozmorov*

* **Correspondence:** Mikhail Dozmorov, mikhail.dozmorov@vcuhealth.org

Supplementary Table 1. Select data science resources. Metrics in all tables were assessed on 2018-11-30.

Name	Description	URL	Stars	Watchers	Forks
free-programming-books	books Freely available programming books	https://github.com/EbookFoundation/free-programming-books	114409	8014	28871
every-programmer-should-know	A collection of mostly technical things every software developer should know	https://github.com/mtdvio/every-programmer-should-know	35592	1652	3201
awesome-public-datasets	A topic-centric list of HQ open datasets in public domains New PR	https://github.com/awesomedata/awesome-public-datasets	29399	1901	4882
Best-websites-a-programmer-should-visit	link Some useful websites for programmers	https://github.com/sdmg15/Best-websites-a-programmer-should-visit	23387	986	2587
awesome-docker	whale A curated list of Docker resources and projects	https://github.com/veggiemonk/awesome-docker	13016	667	1493
awesome-R	A curated list of awesome R packages frameworks and software	https://github.com/qinwf/awesome-R	3260	389	1105
awesome-pipeline	A curated list of awesome pipeline toolkits inspired by Awesome Sysadmin	https://github.com/pditommaso/awesome-pipeline	1793	147	211
awesome-rshiny	An awesome R-shiny list	https://github.com/grabear/awesome-rshiny	207	27	48

Supplementary Table 2. Examples of lists of lists of computer science and machine learning resources.

Name	Description	URL	Stars	Watchers	Forks
awesome	sunglasses Curated list of awesome lists	https://github.com/sindresorhus/awesome	97234	5919	12915
awesome-machine-learning	A curated list of awesome Machine Learning frameworks libraries and software	https://github.com/josephmisiti/awesome-machine-learning	36680	3162	9052
awesome-courses	books List of awesome university courses for learning Computer Science	https://github.com/prakhar1989/awesome-courses	26568	2192	5477
awesome-awesomeness	A curated list of awesome awesomeness	https://github.com/bayandin/awesome-awesomeness	22783	1706	2904
awesome-deep-learning	A curated list of awesome Deep Learning tutorials projects and communities	https://github.com/ChristosChristofidis/awesome-deep-learning	10609	1130	3201
awesome-awesome	A curated list of awesome curated lists of many topics	https://github.com/emijrp/awesome-awesome	1240	125	170
mlr	mlr Machine Learning in R	https://github.com/mlr-org/mlr	1121	102	317

Supplementary Table 3. Impact metrics of popular bioinformatics tools and resources. Only software that is being developed on GitHub, has over 50 stars, and published in peer-review journals was selected.

Name	Description	GitHub	Stars	Watchers	Forks	DOI	Journal	Year	Altmetrics	Impact Factor	CiteScore	Citations
samtools	Tools written in C using htslib for manipulating next-generation sequencing data	https://github.com/samtools/samtools	679	110	366	10.1093/bioinformatics/btp352	Bioinformatics	2009	72.530	5.481	7.84	12191

bwa	Burrow-Wheeler Aligner for short-read alignment see minimap2 for long-read alignment	https://github.com/lh3/bwa	613	118	321	10.1093/bioinformatics/btp324	Bioinformatics	2009	43.358	5.481	7.84	11185
STAR	RNA-seq aligner	https://github.com/alexdobin/STAR	581	89	201	10.1093/bioinformatics/bts635	Bioinformatics	2012	95.740	5.481	7.84	3491
ranger	A Fast Implementation of Random Forests	https://github.com/imbs-hl/ranger	359	42	94	10.18637/jss.v077.i01	Journal of Statistical Software	2016	47.350	22.737	16.32	47
trinityrnaseq	Trinity RNA-Seq de novo transcriptome assembly	https://github.com/trinityrnaseq/trinityrnaseq	319	61	193	10.1038/nbt.1883	Nature Biotechnology	2011	40.096	35.724	12.94	5436
seurat	R toolkit for single cell genomics	https://github.com/satijalab/seurat	308	55	202	10.1038/nbt.4096	Nature Biotechnology	2018	318.540	35.724	12.94	56
MACS	MACS – Model-based Analysis of ChIP-Seq	https://github.com/taoliu/MACS	270	52	168	10.1186/gb-2008-9-9-r137	Genome Biology	2007	15.000	13.214	12.66	3219
canu	A single molecule sequence assembler for genomes large and small	https://github.com/marbl/canu	253	52	75	10.1101/gr.215087.116	Genome Research	2017	89.850	10.101	11.65	305
gemini	a lightweight db framework for exploring genetic variation	https://github.com/arq5x/gemini	235	46	107	10.1371/journal.pcbi.1003153	PLoS Computational Biology	2013	38.984	3.955	4.49	120
bowtie2	A fast and sensitive gapped read aligner	https://github.com/BenLangmead/bowtie2	200	30	70	10.1038/nmeth.1923	Nature Methods	2012	77.088	26.919	13.07	8386
vcftools	A set of tools written in Perl and C for working with VCF files such as those generated by the 1000 Genomes Project	https://github.com/vcftools/vcftools	198	27	84	10.1093/bioinformatics/btr330	Bioinformatics	2011	30.080	5.481	7.84	1864
sga	de novo sequence assembler using string graphs	https://github.com/jts/sga	184	34	74	10.1101/gr.126953.111	Genome Research	2011	36.756	10.101	11.65	312
velvet	Short read de novo assembler using de Bruijn graphs	https://github.com/dzerbino/velvet	182	24	75	10.1371/journal.pone.0008407	PLoS ONE	2009	6.500	2.766	3.01	119
hisat2	Graph-based alignment Hierarchical Graph FM index	https://github.com/infphilo/hisat2	182	40	56	10.1038/nmeth.3317	Nature Methods	2015	53.416	26.919	13.07	898
bcftools	This is the official development repository for BCFtools To compile the develop branch of htslib is needed git clone –branchdevelop git//githubcom/samtools/htslibgit htslib	https://github.com/samtools/bcftools	180	52	115	10.1093/bioinformatics/btw044	Bioinformatics	2016	8.250	5.481	7.84	28
cufflinks		https://github.com/cole-trapnell-lab/cufflinks	174	41	94	10.1038/nbt.1621	Nature Biotechnology	2010	44.434	35.724	12.94	5203
vcfanno	annotate a VCF with other VCFs/BEDs/tabixed files	https://github.com/brentp/vcfanno	170	21	29	10.1186/s13059-016-0973-5	Genome Biology	2016	10.700	13.214	12.66	12
giggle	Interval data structure	https://github.com/ryanlayer/giggle	159	20	19	10.1038/nmeth.4556	Nature Methods	2018	102.350	26.919	13.07	2
Basset	Convolutional neural network analysis for predicting DNA sequence activity	https://github.com/davek44/Basset	156	22	63	10.1101/gr.200535.115	Genome Research	2016	50.430	10.101	11.65	95
lumpy-sv	lumpy a general probabilistic framework for structural variant discovery	https://github.com/arq5x/lumpy-sv	155	27	74	10.1186/gb-2014-15-6-r84	Genome Biology	2013	42.400	13.214	12.66	217
abyss	microscope Assemble large genomes using short reads	https://github.com/bcgsc/abyss	150	24	69	10.1093/bioinformatics/btp367	Bioinformatics	2009	3.000	5.481	7.84	242
ldsc	LD Score Regression LDSC	https://github.com/bulik/ldsc	147	23	81	10.1038/ng.3404	Nature Genetics	2015	52.310	27.125	21.12	243
mothur	Welcome to the mothur project initiated by	https://github.com/mot	145	33	70	10.1128/aem.015	Applied &	2009	23.250	3.633	3.99	7535

	Dr Patrick Schloss and his software development team in the Department of Microbiology & Immunology at The University of Michigan This project seeks to develop a single piece of open-source expandable software to fill the bioinformatics needs of the microbial ecology community	hur/mothur				41-09	Environmental Microbiology					
delly	DELLY2 Structural variant discovery by integrated paired-end and split-read analysis	https://github.com/dellytools/delly	136	35	62	10.1093/bioinformatics/bts378	Bioinformatics	2012	23.500	5.481	7.84	381
qiime2	Official repository for the QIIME 2 framework	https://github.com/qiime2/qiime2	122	35	82	10.1038/nmeth.f.303	Nature Methods	2010	44.208	26.919	13.07	9982
mummer	Mummer alignment tool	https://github.com/mummer4/mummer	121	22	34	10.1371/journal.pcbi.1005944	PLoS Computational Biology	2018	105.700	3.955	4.49	16
monocle-release		https://github.com/cole-trapnell-lab/monocle-release	112	33	54	10.1038/nbt.2859	Nature Biotechnology	2014	78.296	35.724	12.94	501
HiC-Pro	HiC-Pro An optimized and flexible pipeline for Hi-C data processing	https://github.com/nservant/HiC-Pro	101	23	71	10.1186/s13059-015-0831-x	Genome Biology	2015	10.800	13.214	12.66	88
clinvar	This repo provides tools to convert ClinVar data into a tab-delimited flat file and also provides that resulting tab-delimited flat file	https://github.com/macarthur-lab/clinvar	98	42	43	10.1093/nar/gkx1153	Nucleic Acids Research	2017	13.530	11.561	10.84	39
ballgown	Bioconductor package ballgown devel version Isoform-level differential expression analysis in R	https://github.com/alyssafrazee/ballgown	95	23	49	10.1038/nbt.3172	Nature Biotechnology	2015	47.508	35.724	12.94	67
DanQ	A hybrid convolutional and recurrent neural network for predicting the function of DNA sequences	https://github.com/uci-cbcl/DanQ	94	20	43	10.1093/nar/gkw226	Nucleic Acids Research	2016	3.250	11.561	10.84	52
stringtie	Transcript assembly and quantification for RNA-Seq	https://github.com/gpertea/stringtie	90	19	24	10.1038/nprot.2016.095	Nature Protocols	2016	81.946	12.423	10.98	208
scLVM	scLVM is a modelling framework for single-cell RNA-seq data that can be used to dissect the observed heterogeneity into different sources thereby allowing for the correction of confounding sources of variation	https://github.com/PMBio/scLVM	83	23	38	10.1038/nbt.3102	Nature Biotechnology	2015	172.016	35.724	12.94	326
CNVnator	a tool for CNV discovery and genotyping from depth-of-coverage by mapped reads	https://github.com/abyzovlab/CNVnator	76	12	33	10.1101/gr.114876.110	Genome Research	2011	27.836	10.101	11.65	429
SIMLR	Implementations in both Matlab and R of the SIMLR method The manuscript of the method is available at https//wwwnaturecom/articles/nmeth4207	https://github.com/BatzoglouLabSU/SIMLR	65	18	36	10.1038/nmeth.4207	Nature Methods	2017	47.250	26.919	13.07	42
SnpEff		https://github.com/pcingola/SnpEff	64	22	38	10.4161/fly.19695	Fly	2014	9.500	1.218	1.27	1785
Artemis	Artemis is a free genome viewer and annotation tool that allows visualization of sequence features and the results of analyses within the context of the sequence and its six-frame translation	https://github.com/sanger-pathogens/Artemis	63	13	33	10.1093/bioinformatics/btr703	Bioinformatics	2011	26.334	5.481	7.84	271

MAST	Tools and methods for analysis of single cell assay data in R	https://github.com/RGLab/MAST	62	9	28	10.1186/s13059-015-0844-5	Genome Biology	2015	48.806	13.214	12.66	126
ZIFA	Zero-inflated dimensionality reduction algorithm for single-cell data	https://github.com/epierson9/ZIFA	61	8	23	10.1186/s13059-015-0805-z	Genome Biology	2015	41.676	13.214	12.66	95
svtyper	Bayesian genotyper for structural variants	https://github.com/hall-lab/svtyper	61	11	26	10.1038/nmeth.3505	Nature Methods	2015	152.438	26.919	13.07	58
deconstructSigs	deconstructSigs	https://github.com/raerose01/deconstructSigs	60	10	19	10.1186/s13059-016-0893-4	Genome Biology	2016	22.650	13.214	12.66	120
sciclone	An R package for inferring the subclonal architecture of tumors	https://github.com/genome/sciclone	59	48	35	10.1371/journal.pcbi.1003665	PLoS Computational Biology	2014	31.912	3.955	4.49	103
htseq	HTSeq is a Python library to facilitate processing and analysis of data from high-throughput sequencing HTS experiments	https://github.com/simon-anders/htseq	59	9	37	10.1093/bioinformatics/btu638	Bioinformatics	2014	55.346	5.481	7.84	3084
circlator	A tool to circularize genome assemblies	https://github.com/sanger-pathogens/circlator	58	17	20	10.1186/s13059-015-0849-0	Genome Biology	2015	50.358	13.214	12.66	116
clonevol	Inferring and visualizing clonal evolution in multi-sample cancer sequencing	https://github.com/hdng/clonevol	56	8	25	10.1093/annonc/mdx517	Annals of Oncology	2017	15.650	13.926	8.97	7
methylKit	R package for DNA methylation analysis	https://github.com/al2na/methylKit	55	15	64	10.1186/gb-2012-13-10-r87	Genome Biology	2011	23.350	13.214	12.66	283
PhenoGraph	Subpopulation detection in high-dimensional single-cell data	https://github.com/jacoblevine/PhenoGraph	53	7	25	10.1016/j.cell.2015.05.047	Cell	2015	32.588	31.398	21.99	217
TADbit	TADbit is a complete Python library to deal with all steps to analyze model and explore 3C-based data With TADbit the user can map FASTQ files to obtain raw interaction binned matrices Hi-C like matrices normalize and correct interaction matrices identify and compare the so-called Topologically Associating Domains TADs build 3D models from the interaction matrices and finally extract structural properties from the models TADbit is complemented by TADkit for visualizing 3D models	https://github.com/3DGenomes/TADbit	51	15	48	10.1371/journal.pcbi.1005665	PLoS Computational Biology	2017	18.400	3.955	4.49	22
weblogo	WebLogo 3 Sequence Logos redrawn	https://github.com/WebLogo/weblogo	51	10	20	10.1101/gr.849004	Genome Research	2004	9.500	10.101	11.65	4467
targetfinder		https://github.com/shwhalen/targetfinder	50	10	15	10.1038/ng.3539	Nature Genetics	2016	194.740	27.125	21.12	79
