# Supplementary figures and images for: GitHub Statistics as a Measure of the Impact of Open-Source Bioinformatics Software
Source: Front Bioeng Biotechnol. 2018 Dec 18;6:198. doi: 10.3389/fbioe.2018.00198 (PMC6306043; doi:10.3389/fbioe.2018.00198)

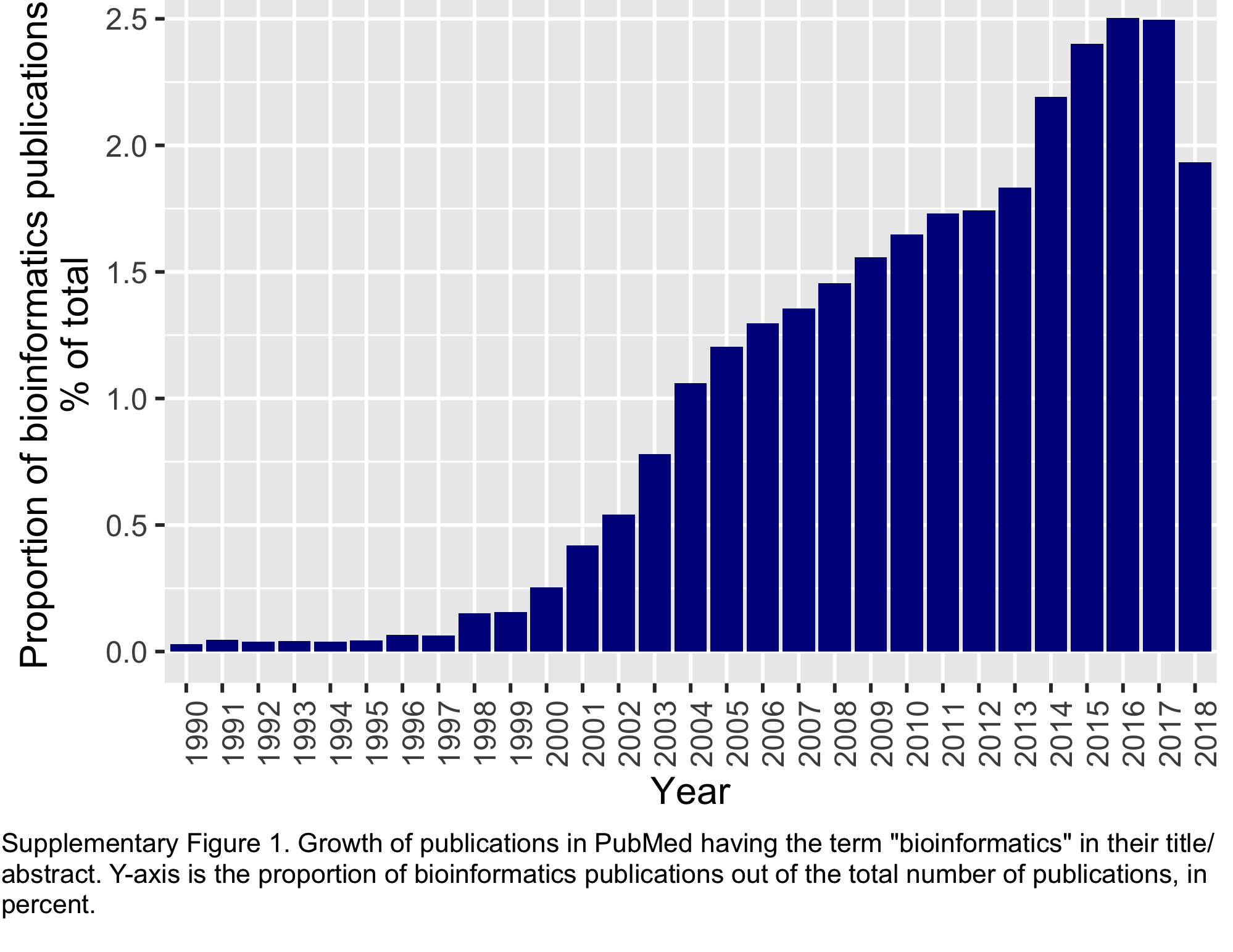

Supplement: Supplementary file 2 [file Image_1.TIFF]

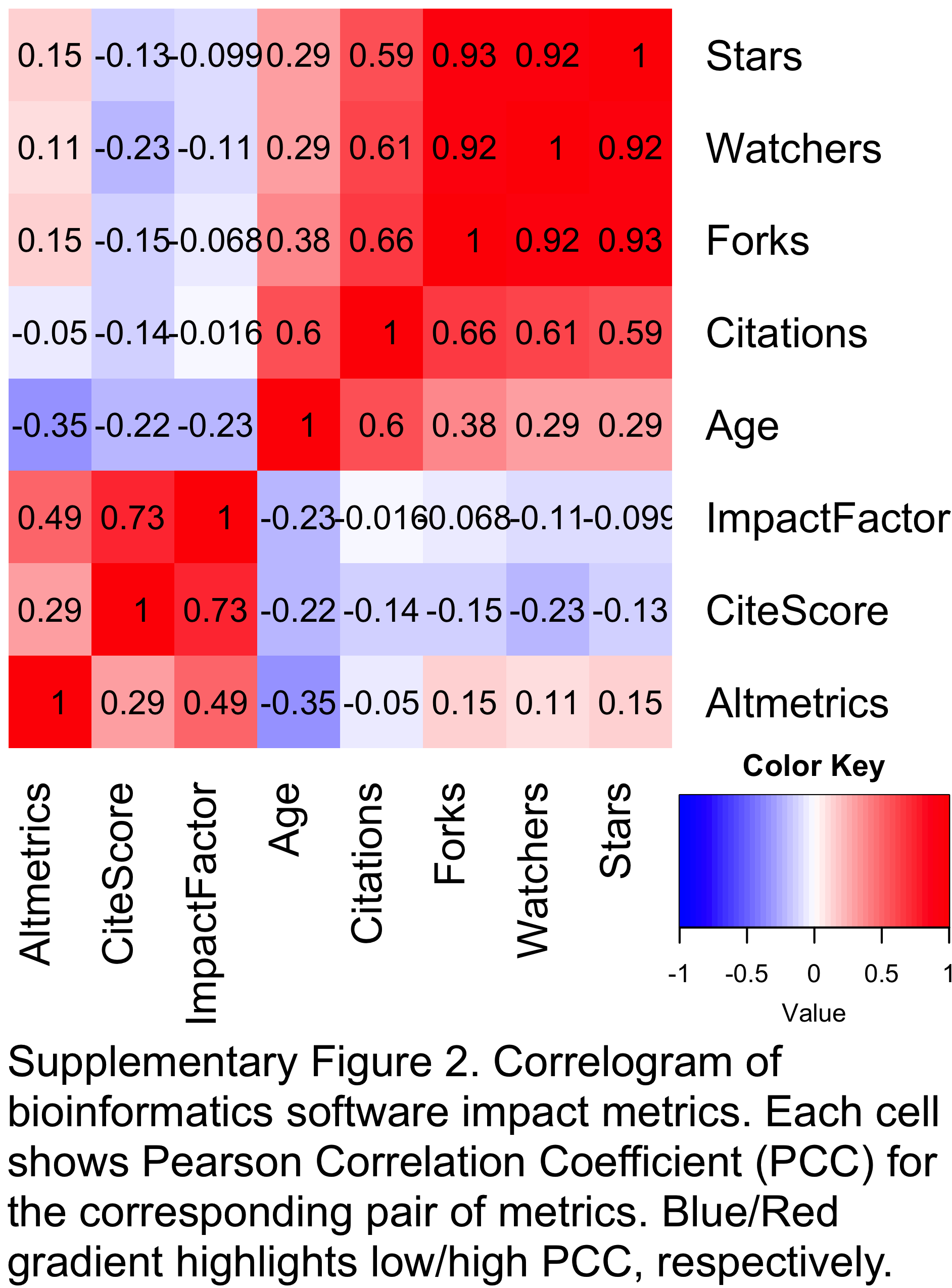

Supplement: Supplementary file 3 [file Image_2.TIFF]
